# Promotion of physical activity-related health competence using digital workplace-based health promotion: a pilot study for office workers

**DOI:** 10.3389/fpubh.2025.1437172

**Published:** 2025-01-30

**Authors:** Leonard Oppermann, Marie-Luise Dierks

**Affiliations:** Department for Patient Orientation and Health Education, Institute for Epidemiology, Social Medicine and Health Systems Research, Hannover Medical School, Hannover, Germany

**Keywords:** physical activity-related health competence, physical activity, health promotion, health literacy, workplace, office workers, pilot study

## Abstract

**Introduction:**

Engaging in health-enhancing physical activity (HEPA) can reduce the risk of developing chronic diseases, which is particularly important for office workers with sedentary lifestyles. Therefore, time- and location-independent interventions for increasing HEPA are necessary.

**Methods:**

To achieve long-term changes in HEPA, interventions can be based on physical activity-related health competence (PAHCO). 48 office workers (83% female, 50 ± 8 years) completed an intervention consisting of bi-weekly exercise videos for 5 weeks, supplemented by PAHCO and anatomical education. The participants’ HEPA levels were measured using the Physical Activity, Exercise, and Sport Questionnaire (Bewegungs- und Sportaktivität Fragebogen; BSA-F)and a physical activity diary, with follow-up measurements at 3 months.

**Results:**

There was a significant increase in PAHCO (*p* = 0.002), especially in control competence (*p* < 0.001), after the intervention and at follow-up. The other sub-competences also increased, but not significantly. HEPA decreased after the intervention and at follow-up, but the decrease was not statistically significant.

**Discussion:**

PAHCO increases after the end of the intervention, especially through the sub-competence of control competence. The other two sub-competences also improved, but not significantly. Participating in the study had no impact on HEPA as an outcome of the PAHCO model. Our study provides preliminary evidence that PAHCO can be enhanced through digital, time- and location-independent interventions. Future research should utilize a randomized controlled design to be able to causally attribute the effects of PA interventions in office workers to the intervention and objective measurements for HEPA should be employed.

## 1 Introduction

Almost half of the population in Germany has at least one chronic disease (1). Physical activity (PA) can be a protective factor in preventing these diseases: The risk of developing chronic diseases such as stroke, hypertension, colon and breast cancer, type 2 diabetes and cardiovascular disease is reduced by 20–30% if World Health Organizations (WHO) recommendations for PA are met (2). The WHO (3) recommends engaging in 150 min or more of PA per week, although any PA is better than none. However, individuals who transition from being completely sedentary to engaging in any health-enhancing physical activity (HEPA) experience the greatest benefits in terms of health outcomes. This is the case even if they have not yet met the WHO’s recommended amount (2, 4). In Germany, 44.8% of women and 51.2% of men meet the WHO recommendations, the proportion tends to decrease with age (5).

One contributing factor for low PA levels is seen in the increased sitting time, especially sitting time at work: individuals spend an average of 207 min sitting at work per day, which accounts for 33% of total sitting time. Among these workers, 35% do not sit at work at all or for a maximum of 1 h, while 19% sit for over 6 h (6). People who work from home partially or entirely tend to spend more time sitting than those who do not (7). According to Techniker Krankenkasse (8), 42% of individuals report predominantly sedentary work.

In order to address the issue of prolonged sitting and its associated health risks, it is necessary to implement PA-interventions in the workplace. This offers a chance for office workers to incorporate PA into their daily work routine. It has previously been observed that a high proportion of them fail to meet the recommended levels of PA and spend prolonged periods of work time sedentary (9, 10). Workplace exercise interventions may increase PA levels (11). Nevertheless, it remains uncertain whether they also have a positive impact on health (12, 13). Observational studies showed that leisure time PA positively affects health. In contrast, high occupational PA may not provide any benefits, and can even have negative impacts on health (14). However, the WHO regards the workplace as a particularly important setting for adults to increase their physical activity (15).

One possible approach to addressing this so-called PA paradox could be to use the model of physical activity-related health competence (PAHCO) as the theoretical framework for developing PA interventions. The model consists of three sub-competences. Movement competence contains the physical level of competence, e.g., motor abilities and skills to perform physical activities both in planned exercise and in everyday activities. Control competence ensures to make correct decisions regarding the amount and intensity of PA to improve biopsychosocial health. And self-regulation competence contains volitional and motivational abilities to ensure regular PA (16). Confirmatory factor analyses were used to distinguish between 10 different factors that form the three sub-competences (17).

While PAHCO is delivered through PA at work, it is reasonable to assume that the effects extend to other areas of the participants’ lifes, resulting in positive health effects from the PA at work. Interventions have demonstrated the ability to produce temporary stable changes in PAHCO, leading to positive influence on PA. As such, the PAHCO model is suitable for planning PA interventions (18).

The model has been used in several interventional studies, including pupils and individuals with intellectual disabilities (19, 20). Recently, Blaschke et al. (18) conducted an intervention focusing on HEPA and PAHCO in office workers as part of a three-week workplace health promotion program. The program consisted of different components: a physical examination and consultation with a physician, guided training with an exercise therapist, lectures and workshops on HEPA with a psychologist and individual training. There was a significant increase in PAHCO immediately after the intervention, which did not significantly decrease after two follow-up measurements up to 18 months after the intervention.

Nonetheless, there is a requirement for relatively short, time- and location-independent interventions to reach participants independently of factors as remote work, meetings and other appointments. In addition, outreach programs for physical activity promotion may potentially reach more employees and may be easier to integrate into regular work routines (21). Furthermore, behaviors learned in a real-world setting may be more readily integrated in normal working life after the study has ended.

The aim of the present pilot study was to develop and evaluate a five-week video-based intervention including videos with exercises that can be done directly at the individual desk, aspects of PAHCO and anatomical knowledge and assess its effects on HEPA in office workers.

## 2 Materials and methods

### 2.1 Study design and participants

The study was planned as a controlled before-and-after study, with one company serving as the intervention group and one as the control group. The allocation was randomized. The trial has been registered with the DRKS register (DRKS00028053) and has received ethical approval from the Hannover Medical School Ethics Committee (Nr. 10157_BO_K_2022). Participants signed a written informed consent before participation.

The study protocol has been published elsewhere (22). Unfortunately, recruitment for the control group did not follow the intended procedures. As a result, the number of participants was insufficient for statistical analysis. This deviation from the protocol led to the study being conducted as a non-controlled pilot in a single financial sector company based in Hannover (Lower Saxony, Germany). The inclusion criteria for the study were: Participants had to be between the ages of 18 and 67 and have a predominantly sedentary primary job. Participants were excluded if they were incapable of giving consent, were pregnant, have had surgery 6 weeks before the start of the study, or were not allowed to exercise due to a physician’s prescription.

### 2.2 Intervention description

The intervention description is based on the Consensus on Exercise Reporting Template (CERT (23); see Supplementary material 1 for the checklist). The intervention group received two weekly videos via email for 5 weeks. The intervention period was relatively brief, largely as a consequence of economic constraints. Nevertheless, its duration was also deliberately limited, with the objective of stimulating an active and healthy lifestyle based on the PAHCO model. The videos demonstrated an exercise that can be done directly at the individual desk. The first exercise was consistent for all participants each week. For the second exercise, participants were given two options to select based on their preferences, enabling the intervention to be tailored to individual needs. The participants completed the exercises individually, in their preferred location, but with the possibility to do them together with colleagues in the office. There was no supervision of the exercises, but a chat room was available for participants to ask the exercise instructor any questions they had.

The exercises were deliberately chosen to be manageable for all participants in their basic variant and easy to learn. For all exercises, however, optional variations were given that are more challenging so that the participants could improve throughout the intervention. Participants were encouraged to progress the exercises themselves. They received information on the FITT (frequency, intensity, time, type) principle during the first week of the intervention. The exercises targeted different body areas. One focus was on strengthening and stretching the shoulder and neck muscles, as these areas are particularly strained when sitting (24), another focus was to target large muscle groups to improve cardiovascular fitness.

Examples of the exercises were sit-ups to strengthen the abdominal muscles while sitting on a chair, reverse butterflies with water bottles in the hands to strengthen the shoulder and neck muscles, or an exercise to stretch the chest, shoulder and neck muscles (see Supplementary material 2 for all exercises). Participants needed a broomstick, a chair and two bottles filled with water to be able to do every exercise. Over 5 weeks of intervention, participants received 10 exercises. All exercise videos remained accessible over the study period.

In the videos, the exercise is briefly shown by three different models (different age and gender). Then the muscles involved and their function are explained using anatomical drawings. This is followed by a step-by-step explanation of the exercises, advice on the number of repetitions and frequency of training (2–3 sets with 10–12 repetitions for resistance exercises, 3 sets with 30 s exercise and 10 s rest for endurance exercises and no limits for stretches), as well as showing alternatives (e.g., sit-ups on the floor, reverse butterflies while standing, bouncing in the final position of the stretch). The exercise instructor holds a M.A. in sport and exercise science. The next section in the video explains a specific aspect of the model of PAHCO (e.g., “What is strength training? How do I assess my sense of exertion? What influence do muscles have on posture?”). Each of the 10 PAHCO factors was covered theoretically in a video and linked to the exercise. For explanation, short video sequences, animated clips and practical examples are part of the intervention. The videos last about 5 mins.

Inspired by the model of PAHCO, the participants were encouraged to reflect on the intensity of the exercise, the number of repetitions and the frequency of training and to put them together according to their preferences to remain active in a health-promoting way independently even after the end of the study. They were encouraged to exercise as much as they want, any type they want.

The PAHCO model assumes that the three levels of physical exercise, learning and experience should be addressed simultaneously in a training session to increase PAHCO (25, 26). The level of physical exercise is conveyed through the exercise, via the visualization and step-by-step explanation. The learning aspect is covered by the accompanying explanations of the muscles involved and their function in everyday life and the specific aspects of PAHCO. The PAHCO aspects also play a role in the experience. In the videos is shown that PA is also suitable for affect regulation. Corresponding exercises, for example to distract one-self from work for a moment and to refocus better afterwards, are shown. The participants can try them out immediately and experience these effects.

In addition, a reflection task is added here, which encourages the participants to relate the aspect to the exercise presented in the video and to their everyday life. As an example, when receiving instructions on maintaining proper posture while sitting at a desk, participants can attempt the tips provided and experience the effects on their posture. Table 1 shows an example of the first week of the intervention.

**Table 1:** Example of the first week of the intervention.

	Exercise	PAHCO-aspect	Physical exercise	Learning	Experience
Video 1	Activation for the shoulder and neck muscles with a broomstick	Self-Control	Upright posture while sitting Mobility of the shoulder muscles	Strategies for self-control Strategies to prevent bad posture and thereby counteract pain caused by sitting	Perception of progress (mobility) Reflection of the posture at the desk
Video 1.1	Sit-ups sitting on a chair	Control of Physical Load	Strengthening of the abdominal muscles	Self-assessment and control of physical load (Borg-Scale and heart rate) Influence of posture on pain, muscle interaction	Influence of breath on muscle tension Self-assessment of physical load
Video 1.2	Hip lift sitting on a chair		Strengthening of the gluteal muscles	Self-assessment and control of physical load (Borg-Scale and heart rate)	Perception of progress (strengthening, more strenuous variations are manageable) Self-assessment of physical load

In addition to the videos, once a day, all participants received an email to remind them about the videos and motivate them at the same time to take a short PA break. The emails referred to the current exercise video and contained a link to a physical activity diary (PA diary) to fill in the PA of the previous day. Daily reminders contained small motivational messages, some related to the exercises (Extend your elbows when you are doing the broomstick activation today!), some conveyed knowledge about PA (Housework and gardening also count as exercise, which can have a positive effect on your health!) or fun facts about animals (Agile frogs jump 35 times their body size from a standing start—how about you?). Participants were provided with a weekly summary of their PA diary, enabling them to monitor their progress.

Before the start of the intervention, participants could choose whether they preferred to receive the reminder in the morning, at noon, or in the afternoon. After the intervention, they were asked how many videos they watched.

### 2.3 Measurements

There were three measurement points in the study: Before the start of the intervention (T0; August/September 2022), directly after the intervention (T1; October 2022) and follow-up after 3 months (T2; January/February 2023). All surveys took place online.

#### 2.3.1 Demographic data

At all three measurement points demographic data was assessed, including age, gender, height and weight and how many days the participants worked from home in the last 4 weeks. Additionally, the educational level was assessed at T0.

#### 2.3.2 Primary outcomes

The primary outcome of the study is the amount of HEPA before and after the intervention. Two data collection methods were utilized to achieve this: the BSA-F (27) was administered at T0, T1, and T2 and a PA diary was kept during the intervention period and the week before T2. The BSA-F was developed to measure self-reported PA with a short, flexible questionnaire and a clear structure. It contains three areas of PA: PA at work, PA during leisure time/transportation and sports activity. For the parts on PA in leisure time/transportation and sports and exercise activity, an index can be formed in the unit minutes per week. The structure of the PA diary is similar to the BSA-F, the participants documented type, duration and intensity of PA on a daily basis. Subsequently, for both the questionnaire and the PA diary, metabolic equivalent (MET) minutes were calculated. BSA-F MET minutes are displayed per week, PA diary MET minutes per day. The appropriate METs according to Ainsworth et al. (28) were assigned to the given activities, multiplied by the duration of the activity and added weekly to obtain the weekly MET minutes. Activities with less than 3.0 METs have too little intensity, so they were excluded as they do not count as HEPA (3). To avoid overestimating its effects, walking was given a weighting factor of 0.5 (29). HEPA was recorded in both ways, as we presumed that the PA diary data was more accurate since it was collected on the day following the activity. However, it was also likely to have more missing data as some participants may have forgotten to fill in the PA diary daily.

#### 2.3.3 Secondary outcomes

Secondary outcomes included the assessment of PAHCO, health-related quality of life (HRQoL), and the intervention evaluation. The PAHCO questionnaire (17) comprises 10 different scales and features 42 items, which measure the three sub-competences of physical activity-related health competence. The evaluation was performed at both the sub-competence level and the general score. HRQoL was assessed with the SF-12, which is a shorter version of the SF-36 health status questionnaire (30). Additionally, at T1, the intervention components, including the videos’ structure and content, PA diary, and intervention homepage, were evaluated. The participants rated each component’s helpfulness on a four-point Likert scale in supporting PA during the intervention period. They were also asked how many videos they had watched (ranging from 0 to 10). This allows to investigate whether the number of videos watched correlates with PA and PAHCO.

### 2.4 Sample size

The initial sample size was calculated on the assumption that a between-group comparison for HEPA would be analyzed (22). The calculated sample size required to measure within-group effects of d = 0.23 with a power of 80% and a significance level of 0.05 would have been 184 participants. We also calculated the post-hoc achieved power for HEPA, operationalized by MET-Minutes. The study achieved a power of 12.8% for the primary outcome. Sample size was calculated using G*Power version 3.1.9.6 (31).

### 2.5 Statistical analysis

Our goal is to examine how HEPA changes after the intervention and in follow-up. To test this, repeated measures ANOVAs were conducted for the METs from the PA diary and for METs and PA minutes from the BSA-F at all measurement points. We used Mauchly’s sphericity test and corresponding corrections in case of sphericity violations and Levene’s test to control for homogeneity of variances. To analyze significant differences between pairs of means in the ANOVA, we used *post-hoc* tests with Bonferroni correction. The significance level was set at 0.05 for all tests. Similar methods were used for the secondary outcomes PAHCO and HRQoL. All analyses were conducted using IBM SPSS Statistics 28 software (IBM, 2021).

Furthermore, bivariate correlations between HEPA, PAHCO, HRQoL and the use of the intervention were considered, as well as evaluation questions on intervention content. This allows for a relatively simple examination of the relationships between the variables and a comparison with the data from Blaschke and colleagues (32). It was hypothesized that the more favorable the intervention was rated and the greater the number of videos watched, the more positive the results would be in terms of both primary and secondary outcomes.

## 3 Results

### 3.1 Participant demographic

A total of 237 participants were recruited via email and advertising in the intervention group company’s intranet. Unfortunately, there was a high dropout rate during the study period. The T1 questionnaire was completed by 73 participants (30.8%), the follow-up-questionnaire by 51 (21.5%). A total of 48 participants (20.3%; 40 female, 8 male) took part in all three measurement points and thus finished the study. Their sociodemographic characteristics at T0 are shown in Table 2. Additionally, sociodemographic characteristics for the control group are shown there. The control group was excluded from further statistical analysis due to insufficient number of participants.

**Table 2:** Demographic data of the participants at T0.

	Intervention group (*N* = 48); Mean	Control group (excluded, *N* = 14); Mean
Gender (female), n (%)	40 (83.3%)	8 (57.1%)
Age (years; SD)	49.69 (8.33)	49.36 (10.92)
BMI (m2/kg; SD)	25.34 (4.35)	25.75 (4.56)
Educational level, n (%)		
Secondary school	16 (33.3%)	2 (14.3%)
High school or similar	32 (66.7%)	12 (85.7%)
Professional qualification, n (%)		
Apprenticeship	22 (45.9%)	3 (21.4%)
University degree	23 (47.9%)	11 (78.6%)
Other	3 (6.3%)	–

### 3.2 Impact of the intervention on the primary and secondary outcomes

Our goal is to examine how HEPA changes after the intervention and in follow-up. To test this, repeated measures ANOVAs were conducted. Table 3 presents the repeated-measures ANOVAs of the primary and secondary outcomes for the intervention group, e.g., physical activity from the PA diary and the BSA-F in MET-minutes and minutes, overall PAHCO score and sub-competence scores as well as SF-12 scores.

**Table 3:** Repeated-measures ANOVAs of the primary and secondary outcome parameters (*N* = 48).

	T0	T1	T2	ANOVA		T0 vs. T1		T0 vs. T2	
	Mean (SD)	Mean (SD)	Mean (SD)	F	ηp^2^	Mean differences (T1–T0)	95% CI for mean differences	Mean differences (T2–T0)	95% CI for mean differences
Total METs PA diary (*n* = 18)	389.94 (282.84)	301.40 (226.34)	312.22 (183.63)	1.75	0.09	−88.54	−252.98, 75.91	−77.72	−210.45, 55.05
Total METs PA diary (median) (*n* = 18)	221.38 (200.59)	236.16 (169.09)	230.00 (226.02)	0.05	0.00	14.78	−134.10, 163.66	8.62	−117.91, 135.16
Total METs BSA-F	2561.82 (2250.31)	2424.05 (1758.58)	2127.45 (1998.64)	0.93	0.02	−137.73	−777,03, 501.49	−434.33	−1382.07, 513.40
METs Leisure Time/Transportation	1910.90 (1988.36)	1640.10 (1704.88)	1464.55 (1863.89)	1.24	0.03	−270.80	−818.94, 277.35	−446.34	−1262.67, 369.99
METs Sports & Exercise	650.93 (934.87)	783.95 (854.91)	662.93 (824.59)	0.81	0.02	133.02	−206.08, 472.13	12.01	−285.59, 309.61
Total Minutes per week BSA-F	733.34 (788.72)	694.46 (648.18)	618.78 (733.09)	0.48	0.01	−38.88	−265.00, 187.24	−114.56	−452.72, 223.60
Minutes per week Leisure Time/ Transportation	617.43 (774.24)	529.56 (624.96)	495.45 (718.66)	0.64	0.01	−87.80	−300.88, 125.13	−121.98	−442.74, 198.77
Minutes per week Sports & Exercise	115.91 (139.58)	164.90 (148.37)	123.33 (117.20)	4.26*	0.08	48.99	0.37, 98.36	7.42	−37.58, 52.42
Overall PAHCO score	2.74 (0.55)	2.90 (0.56)	2.93 (0.53)	6.66*	0.12	0.16	0.01, 0.31	0.19	0.05, 0.34
Movement Competence	2.95 (0.54)	2.97 (0.59)	3.05 (0.54)	2.01	0.04	0.02	−0.12, 0.17	0.10	−0.05, 0.25
Control Competence	2.46 (0.73)	2.79 (0.69)	2.83 (0.69)	13.40**	0.22	0.32	0.14, 0.51	0.36	0.16, 0.57
Self-regulation Competence	2.80 (0.60)	2.93 (0.57)	2.92 (0.55)	3.2	0.05	0.13	−0.05, 0.31	0.12	−0.04, 0.27
SF-12 Physical	48.12 (4.47)	49.18 (4.23)	48.74 (3.97)	1.23	0.03	1.06	−0.71, 2.83	0.62	−1.05, 2.29
SF-12 Mental	29.24 (9.19)	26.70 (9.12)	29.04 (10.09)	3.41*	0.07	−2.54	−5.02, −0.07	−0.20	−2.84, 2.44

*p < 0.05, **p < 0.001; Huynh-Feldt correction was used when the data violated the assumption of sphericity.

#### 3.2.1 Physical activity

. The repeated-measures ANOVAs showed significant differences for Sports & Exercise PA Minutes per week. The effect was significant (*F*(2, 94) = 4.26, *p* = 0.017, ηp^2^ = 0.08). Post-hoc pairwise comparisons with a Bonferroni-correction adjustment indicated that Sports & Exercise PA minutes were significantly lower at T2 than at T1 (*p* = 0.039, *d* = 0.37). There was no significant difference between T0 and T1 (*p* = 0.052) and T0 and T2 (*p* = 1.0). There were no significant differences for Total METs, Leisure Time/Transportation METs and Sports & Exercise METs as well as on Total PA Minutes per week and Leisure Time/Transportation PA minutes per week (Table 3).

Additionally, PA was assessed via PA diary. Neither the mean (*F*(2, 33) = 1.75, *p* = 0.189, ηp^2^ = 0.09) nor the median (*F* (2, 33) = 0.05, *p* = 0.955, ηp^2^ = 0.00) changed significantly.

#### 3.2.2 PAHCO

A repeated-measures ANOVA was performed to evaluate the effect of the intervention on the overall PAHCO score (Table 3). The effect of the intervention on the overall PAHCO score was significant at the 0.05 level (*F*(2, 94) = 6.66, *p* = 0.002, ηp^2^ = 0.12). Post-hoc pairwise comparisons with a Bonferroni-correction adjustment indicated that the overall PAHCO score was significantly higher at T1 than at T0 (*p* = 0.033, *d* = 0.38) and similarly at T2 than at T0 (*p* = 0.006, *d* = 0.47), and there was no significant difference between T1 and T2 (*p* = 1.0). That finding indicates, that PAHCO remains constant after the intervention.

Similar analyses were performed on the sub-competence level (Table 3). For movement competence, Mauchly’s test indicated that the assumption of sphericity had been violated (χ^2^ (2) = 8.51, *p* = 0.014.) and therefore degrees of freedom were corrected using Huynh-Feldt estimates of sphericity (*ε* = 0.89). There was no significant effect (*F*(1.77, 83.16) = 2.01, *p* = 0.146, ηp^2^ = 0.04). Similar results can also be observed for self-regulation competence, the effect was not significant (*F*(2, 94) = 2.47, *p* = 0.090, ηp^2^ = 0.05). The effect of the intervention on control competence, was significant (*F*(2, 94) = 13.40, *p* < 0.001, ηp^2^ = 0.22). Post-hoc pairwise comparisons with a Bonferroni-correction adjustment indicated that control competence was significantly higher at T1 than at T0 (*p* < 0.001, *d* = 0.64) and similarly at T2 than at T0 (*p* < 0.001, *d* = 0.63). There was no significant difference between T1 and T2 (*p* = 1.0), which indicates that control competence remains constant over time.

#### 3.2.3 HRQoL

Repeated-measures ANOVAs were performed for the physical and the mental dimension from the SF-12 (Table 3). The effect on the physical dimension was not significant (*F*(2, 94) = 1.23, *p* = 0.297, ηp^2^ = 0.03). There was a significant effect on the mental dimension, (*F*(2, 94) = 3.28, *p* = 0.042, ηp^2^ = 0.07). Post-hoc pairwise comparisons with a Bonferroni-correction adjustment indicated that the mental dimension was significantly lower at T1 than at T0 (*p* = 0.042, *d* = 0.37). There was no significant difference between T0 and T2 (*p* = 1.0) and T1 and T2 (*p* = 0.195).

### 3.3 Evaluation

For the evaluation, the different components of the intervention (Anatomy, Exercises, Health knowledge to go, PA diary, Prompts and Homepage) were rated on their helpfulness on a scale from 1 (not helpful at all) to 4 (very helpful) with 2–5 questions. Means and standard deviations are displayed in Table 4. The most helpful were the Exercises and the explanations on Anatomy, the least helpful were the design of the Homepage and the PA diary, whereby the design of the Homepage was rated by only 22 participants. In a final overall evaluation (Table 5) on a scale from 1 (very good) to 4 (very bad), the Exercises also received the highest score, the PA diary again the lowest.

**Table 4:** Helpfulness of the intervention components (Score 1–4, not helpful at all to very helpful).

Intervention component	N	Mean (SD)
Anatomy	45	3.50 (0.59)
Exercises	47	3.66 (0.46)
Health knowledge to go	40	3.26 (0.40)
PA diary	45	2.84 (0.68)
Prompts	46	3.35 (0.60)
Homepage	22	3.22 (0.35)

**Table 5:** Evaluation of the intervention components (Score 1–4, very good to very bad).

Intervention component	N	Mean (SD)
Exercises	47	1.47 (0.55)
Health knowledge to go	47	1.66 (0.48)
PA diary	44	2.07 (0.55)
Prompts	48	1.58 (0.50)
Homepage	39	1.67 (0.53)

Furthermore, we asked the participants to indicate the number of videos they had watched during the intervention period. The results are presented in Table 6.

**Table 6:** Number of videos watched by the participants.

Number of videos	N
Missing	14
0	1
1–3	2
4–6	7
7–10	24

### 3.4 Relationship between the evaluation and the outcomes

Pearson correlation coefficients were performed to evaluate the relationship between PA and PAHCO and between the evaluation of the study content and PA and between the evaluation of the study content and PAHCO.

There were significant moderate positive correlations between PAHCO overall-score and Sports & Exercise PA Minutes per week for T0 (*r* = 0.39, *p* = 0.006), T1 (*r* = 0.46, *p* = 0.001) and T2 (*r* = 0.39, *p* = 0.006). Similar results can be seen for MET minutes (see Supplementary material 3 for all correlations). There was a significant correlation between the number of watched videos with PAHCO and HEPA at T2: a moderate negative relationship with Leisure Time/Transportation METs (*r* = −0.35, *p* = 0.043) as well as Total METs (*r* = −0.41, *p* = 0.018). We found significant moderate negative correlations at T1 between HRQoL and movement competence (*r* = −0.38, *p* = 0.008) and HRQoL and control competence (*r* = −0.30, *p* = 0.038) and similar at T2 (*r* = −0.33, *p* = 0.021 and *r* = −0.41, *p* = 0.004).

Moderate Pearson correlation coefficients were identified between the evaluation of study content and PA and between the evaluation of the study content and PAHCO at T1, with certain ones displayed here. There is a moderate positive correlation between the helpfulness of the Anatomy (Table 4) and control competence (*r* = 0.304, *p* = 0.042). The overall evaluation of the prompts (Table 5) correlates moderately negatively with Leisure Time/Transportation METs (*r* = −0.370, *p* = 0.010), Leisure Time/Transportation PA Minutes (*r* = −0.354, *p* = 0.014) as well as with total METs (*r* = −0.363, *p* = 0.011) and total PA minutes (*r* = −0.362, p = 0.011). Similar, the overall evaluation of the homepage (Table 5) correlates moderately negatively with Leisure Time/Transportation METs (*r* = −0.344, *p* = 0.032), Leisure Time/Transportation PA Minutes (*r* = −0.341, *p* = 0.034) as well as with total METs (*r* = −0.353, *p* = 0.028) and total PA minutes (*r* = −0.368, *p* = 0.021).

## 4 Discussion

This pilot study aimed to evaluate the effects from a five-week video-based intervention including videos with exercises that can be done directly at the individual desk on HEPA, PAHCO and HRQoL in office workers.

The repeated measures ANOVAs indicate that there is a slight but non-significant decrease in total HEPA after the intervention and at follow-up. In their study, Kyu and colleagues (34) categorize the activity level of the participants into four groups, as follows: below 600 METs is classified as insufficient activity, 600–3,999 METs is classified as low, 4,000–7,999 METs is classified as medium, and above 8,000 METs is classified as high activity level. As the participants in our study showed a decline in activity levels from 2,500 METs at T0 to 2,100 METs at T1, they can still be categorized as having a low but sufficient level of activity. It is also important to acknowledge that the METs in our study were not objectively measured, but rather recorded using retrospective questionnaires. This approach may potentially lead to inaccuracies, given that the recorded period was 4 weeks.

Although there is an initial increase in sports activity in our study, it decreases significantly at follow-up. The results are in line with another study in which 328 office workers underwent a three-week workplace health promotion program based on the PAHCO model (18). The study measured leisure-time physical activity (LTPA) including sports activity, but excluding light PA and active commuting. Post-intervention, LTPA witnessed an initial increase, but it decreased significantly after six and 18 months. However, the study we conducted had less guided HEPA during the intervention period than the study from Blaschke and colleagues, with a minimum of 135 min per day over 3 weeks. Regarding only sports activity, the outcomes show a comparable pattern—an increase after the intervention, followed by a slight decrease during the follow-up period. Another study (33) also showed that self-reported PA increased after an intervention, but then decreased at the six-month follow-up. Nevertheless, a subsequent 13-month follow-up showed an increase in self-reported PA, but not in objectively measured PA. The authors attributed the effect of the first decreasing and then increasing PA to a seasonal pattern and the first follow-up taking place during the winter season. This could be applicable to our research as the three-month follow-up was conducted in January and February, and the questions about cycling to other activities and gardening were accompanied by considerably less PA than in the baseline survey in August/September. A scoping review (35) examining the impact of seasons and weather on PA and other related factors found evidence that people tend to be more active in the summer than in the winter. This finding highlights the need to consider such seasonal variations when designing and implementing PA interventions and interpreting the results. Moreover, it is essential to identify strategies that can maintain PA levels at optimal levels even after the implementation of interventions, besides seasonal pattern.

The total PAHCO score increases significantly with a small effect size after our intervention, which is mainly due to the significant increase in control competence. The effect on control competence can be classified as medium. Movement competence and self-regulation competence increase as well, but not significantly. Our results are consistent with Blaschke’s study (18): PAHCO significantly increased and remained stable after the 18-month follow-up in their intervention. The authors attributed this to the high amount of guided HEPA and the face-to-face design of the intervention. Their design is very different from our study: They conducted at least 135 min of supervised PA per day over 15 days. The amount of supervised PA in our study was much lower, with 10 sessions of 5 min each, and completely digital. Nevertheless, in our study, PAHCO significantly improved, particularly and with a medium effect size in the area of control competence. One reason for this could be that the entire development of the intervention was based on the PAHCO model. This implies that PAHCO was advocated not only by guided HEPA but also by every aspect of the intervention: the videos as the primary intervention content, the PA diary and the daily prompts. The intervention occurring within the day-to-day work of office workers enabled them to practically test and apply the acquired strategies in the relevant context, obviating the need for transfer from an experiment-like environment to everyday life. In another study, aimed at ninth-grade students, Rosenstiel et al. (36) conducted six 90-min Physical Education lessons, in which the content of two scales of the PAHCO model was delivered through running or gameplay interventions. Both theory and practice were incorporated into the sessions. A significant increase in both PAHCO scales was observed in the gameplay intervention group after the intervention, but there were no significant differences at follow-up after 8–12 weeks (20). This demonstrates the similarity with our study that PAHCO can be influenced by interventions. Nonetheless, our research showed that the PAHCO scores remained stable over a longer period of time. Similar outcomes were found by Blaschke et al. (18), who attribute this difference to a lower stability of PAHCO at a younger age.

The mental dimension of HRQoL decreases significantly after our intervention, but returns to almost baseline levels at follow-up. The effect size of the decrease is small and the upper end of the confidence interval is only marginally less than zero, which suggests that the practical significance of the result is likely to be low. These results are in contrast to those of Blaschke et al. (18): immediately after their intervention, HRQoL increases in both dimensions and then decreases slightly after 6 and 18 months, but still remains above the baseline value. The design of our study may account for this difference: while Blaschke and colleagues took a three-week break from work, our intervention was integrated into everyday working life.

The PA diary was considered the least helpful in increasing PA in our study. Self-monitoring, such as keeping a PA diary, is a recognized behavior change technique and listed in Michie’s taxonomy as a helpful intervention component (37). However, a recent meta-analysis highlighted a negative moderating effect of self-monitoring on PA in adults with overweight and obesity (38). Although our study did not investigate the direct association between the PA diary and HEPA, the evaluation of the diary reveals that respondents did not perceive it as positively as other intervention components. Thus, more information is required on how to design and incorporate a PA diary within the intervention to enhance participants’ perceptions of its usefulness.

In our study there were correlations between the total PAHCO score and sports activity, but not Leisure Time/Transportation PA. Blaschke et al. (32) found a positive correlation between the PAHCO scales included in their questionnaire and LTPA, which was not found in our study, except for sports activity. Blaschke and colleagues suggest that an increase in PAHCO leads to an increase in LTPA and thus to an improvement in health in the long term. However, conceptual differences in the measurement of PA may lead to differences in results, as Blaschke and colleagues only measured LTPA and did not include active commuting, for example.

There were positive correlations between control competence and the evaluation of the Anatomy section, but not between control competence and the evaluation of the Health knowledge to go section in our study. The sections on Anatomy and Health knowledge to go were the content of the intervention that most closely addressed control competence. The positive correlation between control competence and the rating for Anatomy supports this, although the absence of correlation with Health knowledge to go contradicts this. However, the measurement focused not on knowledge gain, but on the subjective helpfulness of increased exercise. One method of assessing the impact of health education is to administer a health-related fitness knowledge (HRFK) test, as Volk et al. (39) did with pupils. This tool can demonstrate intervention-related improvements in HRFK among ninth-graders. Nevertheless, it has not been validated with other age groups, so it is not clear whether it is suitable for adult office workers. Moreover, the test comprises 33 items, some of which are open-ended questions, making it relatively long. Volk and colleagues have highlighted that other assessments lack standardized definitions of HRFK, as well as low reliability and validity. However, potential correlations between PAHCO, HEPA and HRFK should be investigated in future studies.

Lastly, there was a positive correlation detected between the scores of the Prompts and the Homepage with Leisure Time/Transportation PA. This suggests that the two components of the intervention provided an encouragement to engage in low-threshold exercise. MacPherson and colleagues (40) found that people at risk of developing type 2 diabetes were more likely to keep their PA diary for up to 3 days after a prompt. Prompts seem to increase awareness of PA, but are not intense enough to have an impact on sports activity. However, this is only a correlation and does not indicate a causal effect.

### 4.1 Strengths and limitations

To the best of our knowledge, this is the first intervention to address PAHCO and HEPA in a real-world setting with office workers. Because of the brief, video-based intervention, participation could be easily assimilated into the everyday work routine. After the intervention, PAHCO increased significantly and remained stable at the higher level 3 months later.

Further strengths arise from our use of the PAHCO questionnaire with all sub-competences, compared to previous studies (18). By using the BSA-F and converting PA minutes to MET minutes, it was also possible to measure HEPA, which is the conceptual outcome of the PAHCO model (17).

However, it is important to note the limitations of this study. The absence of a control group and randomization in the intervention plan means that the causal relationship between the intervention and HEPA and PAHCO cannot be established. Although a control group was initially planned, insufficient participation in the study meant that the results could not be statistically analyzed.

Furthermore, while PA levels were measured using the BSA-F and the PA diary, they were not measured objectively, for example using an accelerometer. To prevent biased evaluations, combined measurement utilizing different methods is advised (41). Unfortunately, only a small number of participants maintained a regular PA diary, therefore the BSA-F was used to assess and analyze PA. However, when evaluating other PA questionnaires, only moderate agreement with objectively measured PA was observed, therefore the findings should be interpreted carefully (42). As the baseline survey was conducted in the late summer and the follow-up survey was conducted in the winter, seasonal effects in PA cannot be excluded, as previously discussed (35). In future studies, PA should therefore be surveyed objectively and at different seasons.

An objective assessment of PA combined with an objective assessment of health outcomes could lead to a better understanding of the PA paradox (14) in the future. It is not possible to conclusively determine whether the PAHCO model is suitable for preventing the PA paradox in workplace interventions, as we did not determine whether the participants engaged in physical activity during their leisure time or at work, but the increase in PAHCO after the intervention is an initial indication that the model is suitable for planning PA interventions in the workplace.

In interpreting the results, it is essential to consider that the implementation of the PA was entirely self-determined and not supervised, which could potentially impact the adherence of the participants and have negative impacts on the correct execution of the exercises. Despite the provision of a chat room for questions, this may have been a more significant barrier than having a personal contact person on site. One potential approach to integrating the benefits of digital technology and the commitment of a face-to-face intervention could be the use of blended interventions (43).

Another limitation of the study is the very high dropout rate, with only 20% of participants completing all questionnaires. Face-to-face interventions have a retention rate of 75% (44), and web-based interventions have a retention rate of 50%, although the range here is very broad at 10–90% (45). A qualitative interview study is currently investigating the reasons for dropout (46).

It should be noted that the majority of participants in the study were female, which may limit the generalizability of the findings to other genders. Despite the small sample size, which did not allow for a comprehensive analysis of gender differences, there were discrepancies in the mean values in some instances. This is a topic that should be the subject of further investigation.

Finally, the study was relatively short in comparison with other workplace PA interventions, with an intervention period of 5 weeks and a follow-up period of 3 months (13). Possible effects on both the HEPA and PAHCO sub-competence movement competence might be better achieved with a longer study duration and a longer follow-up period. This should be taken into account in future studies.

## 5 Conclusion

The study results on the promotion of PAHCO in office workers show that PAHCO increases after the end of the intervention, especially through the sub-competence of control competence. The other two sub-competences also improved, but not significantly. Nonetheless, participating in the study had no impact on HEPA as an outcome of the PAHCO model and no clear effect on HRQoL.

Our study provides preliminary evidence that PAHCO can be enhanced not only through face-to-face or extensive structured PA, but also through digital, time- and location-independent interventions. This provides an important insight for workplace health promotion programs, as PAHCO can be promoted in a time-efficient manner.

Future research should utilize a randomized controlled design to be able to causally attribute the effects of PA interventions in office workers to the intervention. Additionally, to obtain more reliable data on PA, objective measurements such as accelerometers should be employed.

## Data Availability

The raw data supporting the conclusions of this article will be made available by the authors on reasonable request.
