# Supplementary material for: Promotion of physical activity-related health competence using digital workplace-based health promotion: a pilot study for office workers
Source: Front Public Health. 2025 Jan 30;13:1437172. doi: 10.3389/fpubh.2025.1437172 (PMC11821944; doi:10.3389/fpubh.2025.1437172)
Supplement: Supplementary file 1 [file Table_1.DOCX]

**Supplementary Material 1 – CERT Checklist**

1. The participants needed a broomstick, a chair and two bottles filled with water to do the exercises.
2. The exercise instructor holds a M.A. in sport and exercise science.
3. The exercises were performed individually.
4. The exercises were unsupervised and delivered through videos. The participants had the possibility to ask questions to the exercise instructor in a chat.
5. Participants received a daily reminder via email to do an exercise and to fill out a physical activity diary. They could choose the time (in the morning, at noon or in the afternoon) they wanted to be reminded. At T1 they were asked how many videos they watched.
6. The daily reminder contained small motivational messages, some related to the exercises (Extend your elbows when you are doing the broomstick activation today!), some convey knowledge about exercise (Housework and gardening also count as exercise, which can have a positive effect on your health!) or fun facts about animals (Agile frogs jump 35 times their body size from a standing start - what can you do?). The participants received once a week an overview of their physical activity dairy, so they were able to see their progress.
7. The participants were encouraged to progress the exercises themselves. The intervention group received information on the FITT (frequency, intensity, time, type) principle during the first week of intervention.
8. See Supplementary Material 2 for all exercises.
9. The participants were encouraged to exercise as much as they want, any type they want.
10. The videos for the intervention group included elements of PAHCO alongside the exercises. Each of the 10 PAHCO factors was covered theoretically in a video and linked to the exercise.
11. N/A
12. The participants exercised at home or at work, wherever they wanted.
13. The participants received basic information on repetitions and sets for each exercise (2-3 sets with 10-12 repetitions for resistance exercises, 3 sets with 30 seconds exercise and 10 seconds rest for endurance exercises and no limits for stretches).
14. The exercises were self-tailored to participant preferences. The first exercise per week was the same for all participants, for the second they could choose between two exercises to adapt the intervention to their needs. For all exercises, optional variations were given that were more challenging, so that the participants could choose to do them or the basic exercise.
15. All exercises could be performed with different difficulties, but the participants chose for themselves.
16. The participants were asked how many videos they watched during the intervention period. 34 participants answered this question, 14 did not answer. The mean is 7.8 videos, standard deviation is 2.75 and the median is 9 videos.
