# Supplementary material for: Promotion of physical activity-related health competence using digital workplace-based health promotion: a pilot study for office workers
Source: Front Public Health. 2025 Jan 30;13:1437172. doi: 10.3389/fpubh.2025.1437172 (PMC11821944; doi:10.3389/fpubh.2025.1437172)
Supplement: Supplementary file 2 [file Table_2.DOCX]

**Supplementary Material 2 – intervention exercises**

| Week | First exercise per week | Second exercise per week | |
| --- | --- | --- | --- |
| 1 | Activation for the shoulder and neck muscles with a broomstick | Sit-ups sitting on a chair | Hip lift sitting on a chair |
| 2 | Reverse butterfly sitting on a chair | Hamstring stretch | Heel raises and toe lifts |
| 3 | Neck circles | Stretch for the chest, shoulder and neck muscles | Wringing out the towel shoulder stretch |
| 4 | Single-legged squat | Isometric push and pull | Paddling with a broomstick |
| 5 | Row sitting on a chair | Neuromotor finger exercise | Focus on Objects Near and Far with the eyes |
